# Supplementary material for: TrkB-expressing paraventricular hypothalamic neurons suppress appetite through multiple neurocircuits
Source: Nat Commun. 2020 Apr 7;11:1729. doi: 10.1038/s41467-020-15537-w (PMC7138837; doi:10.1038/s41467-020-15537-w)
Supplement: Supplementary file 1 — Supplementary Information [file 41467_2020_15537_MOESM1_ESM.pdf]

TrkB-expressing paraventricular hypothalamic neurons suppress appetite through multiple neurocircuits

Juan Ji An et al.

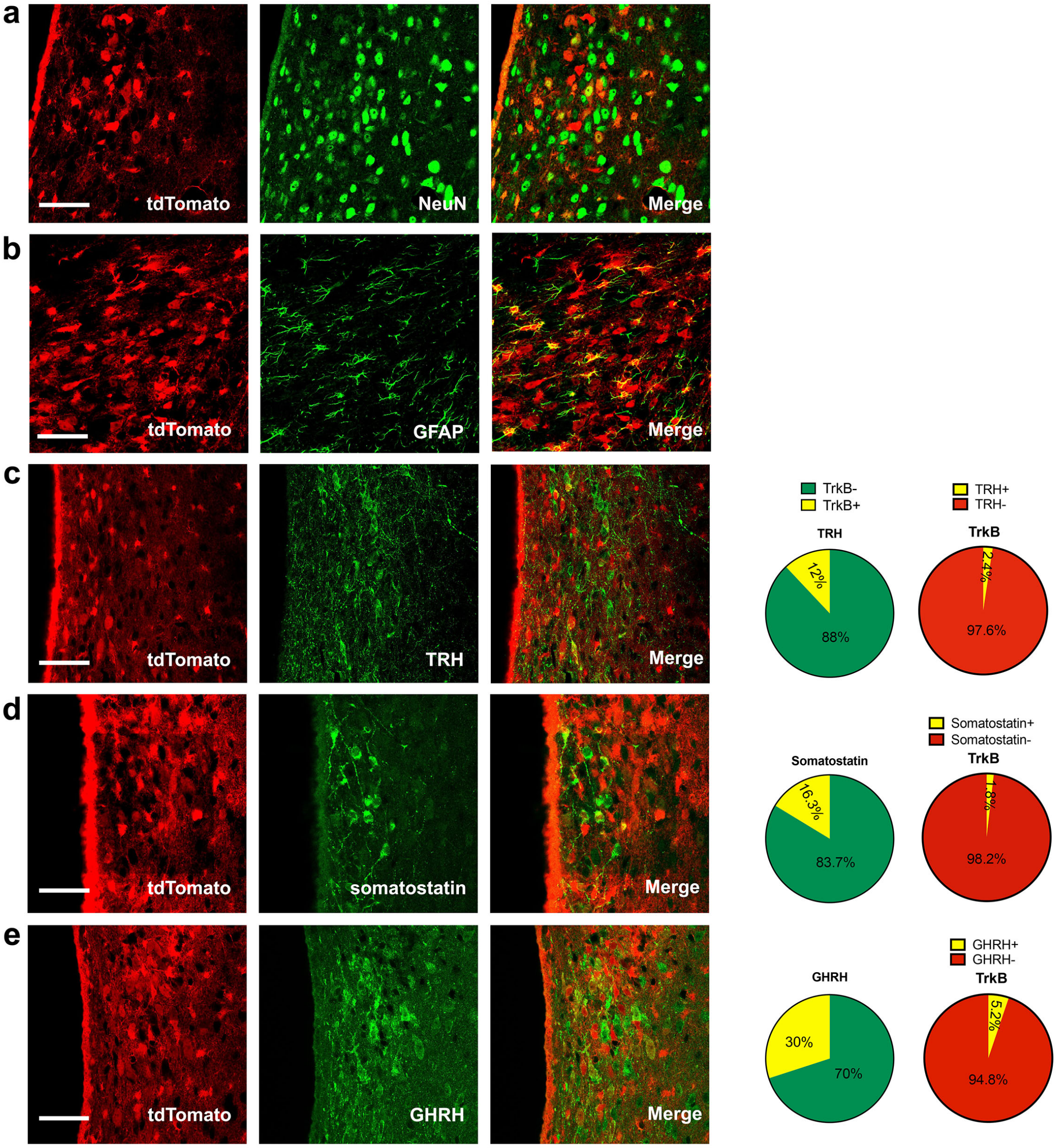

Supplementary Figure 1. Co-expression of TrkB with cellular markers in the PVH. TrkB-expressing cells are marked by tdTomato in tamoxifen-treated *Ntrk2*$^{CreER/+}$*;Rosa26*$^{Ai9/+}$ mice. (**a, b**) The majority of TrkB-expressing cells in the PVH are neurons marked by NeuN, while some of TrkB-expressing cells are GFAP-expressing astrocytes. (**c**-**e**) Some PVH^{TrkB} neurons express TRH, somatostatin, or GHRH. Scale bars are 50 μm long. Source data are provided as a Source Data file.

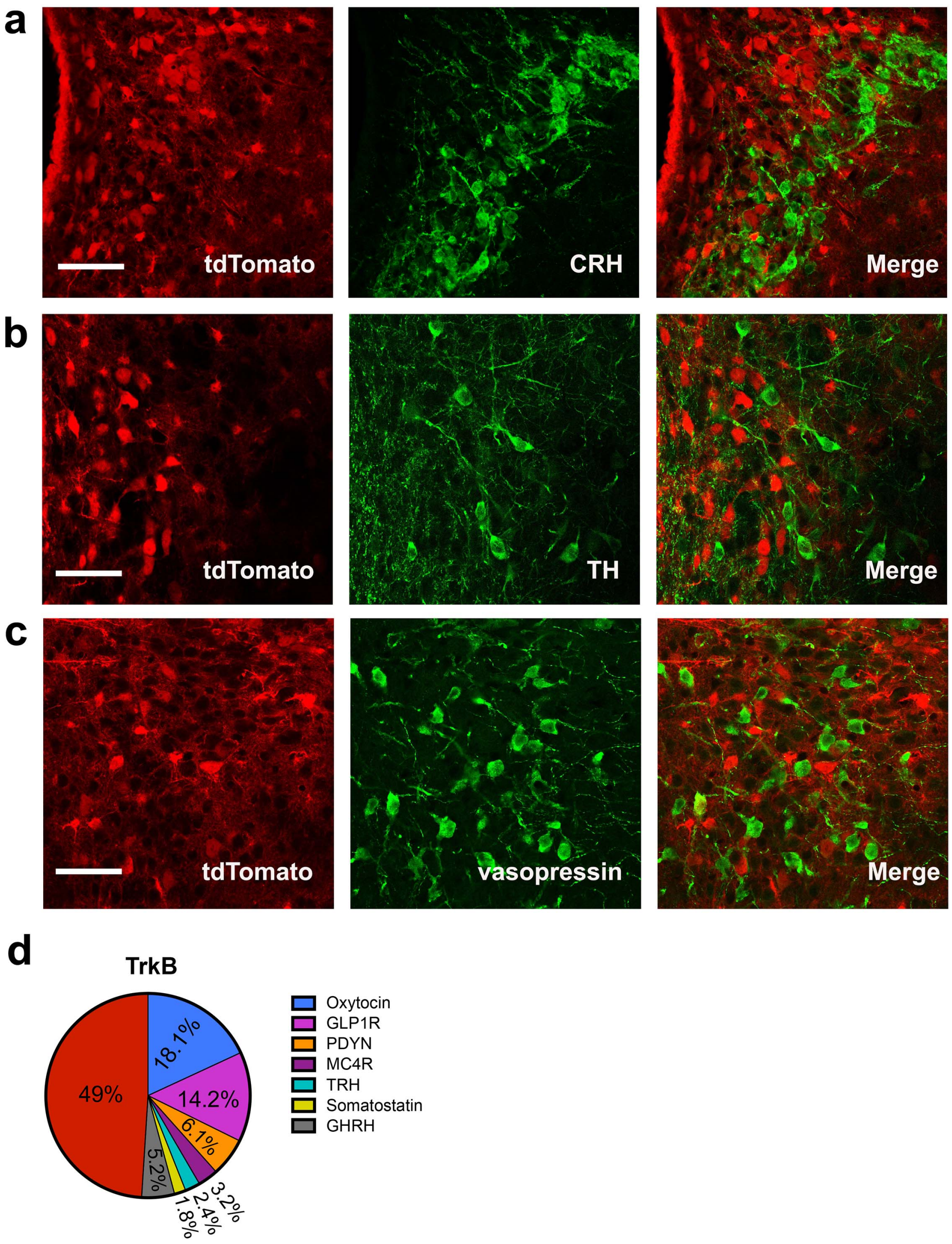

Supplementary Figure 2. Co-expression of TrkB with cellular markers in the PVH. TrkB-expressing cells are marked by tdTomato in tamoxifen-treated *Ntrk2*$^{CreER/+}$*;Rosa26*$^{Ai9/+}$ mice. (**a-c**) Few PVH^{TrkB} neurons express CRH, tyrosine hydroxylase (TH), or vasopressin. Scale bars are 50 μm long. (**d**) Percentage of PVH^{TrkB} neurons that express various markers.

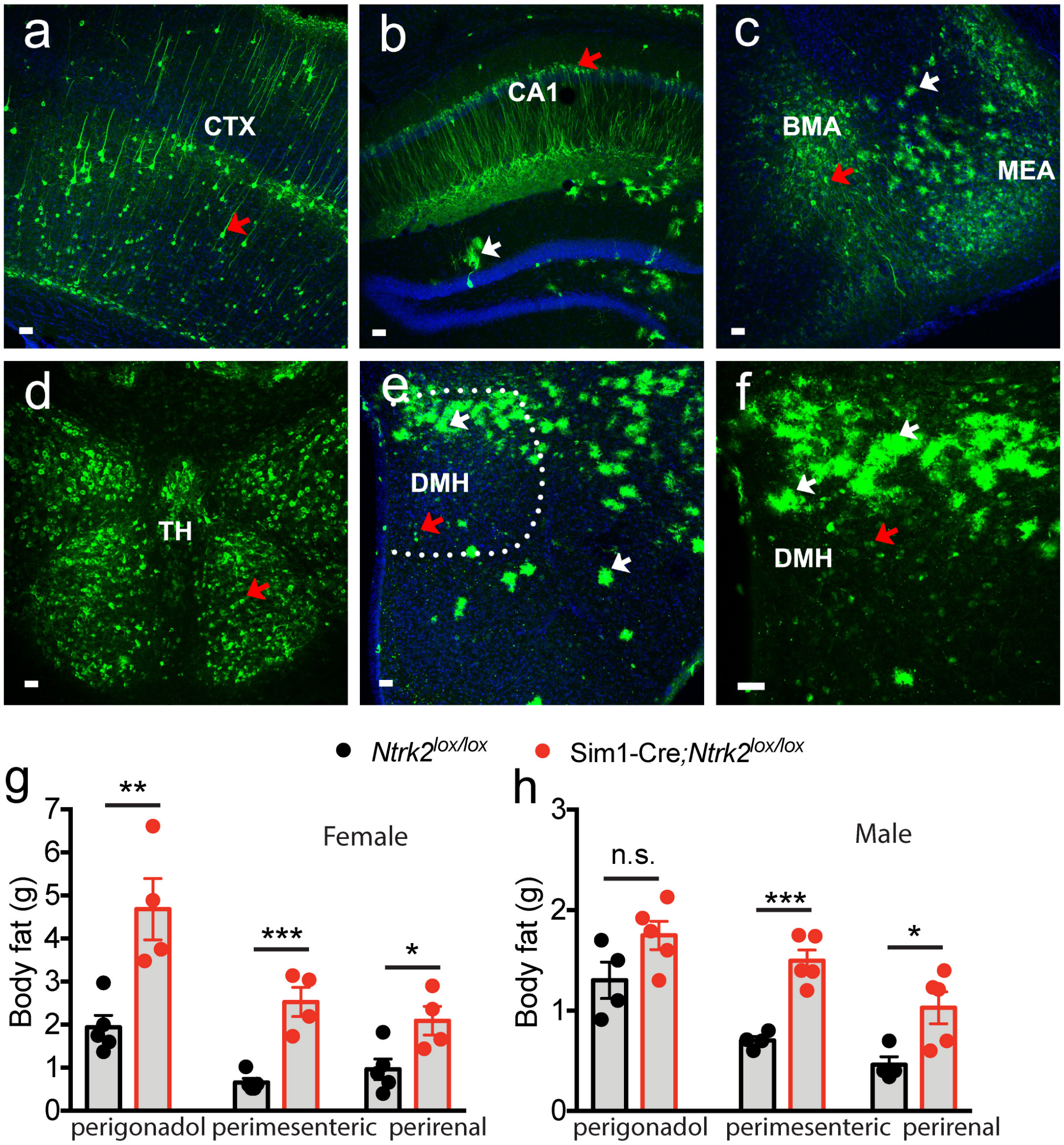

Supplementary Figure 3. Deletion of the *Ntrk2* gene using the Sim1-Cre transgene. (**a-f**) β-galactosidase immunohistochemistry of Sim1-Cre;*Ntrk2*$^{fBZ/+}$ brain sections, showing Cre-mediated deletion of the *Ntrk2*^{fBZ} allele in some TrkB-expressing cells in the cerebral cortex (a), hippocampal CA1 region (b), amygdala (c), thalamus (d), and DMH (e & f). Red and white arrows denote β-galactosidase-expressing neurons and astrocytes, respectively. CTX, cerebral cortex; BMA, basomedial amygdala; MEA, medial amygdala; TH, thalamus; DMH, dorsomedial hypothalamus. Scale bars are 50 μm long. (**g**) Weight of perigonadal, perimesenteric and perirenal adipose tissues in 16-wk-old female *Ntrk2*$^{lox/lox}$ (control) and Sim1-Cre;*Ntrk2*$^{lox/lox}$ (mutant) mice. n = 5 controls and 4 mutants. Two-tailed unpaired *t* test; **P* = 0.026, ***P* = 0.0057, and ****P* = 0.0006. (**h**) Weight of perigonadal, perimesenteric and perirenal adipose tissues in 16-wk-old male *Ntrk2*$^{lox/lox}$ (control) and Sim1-Cre;*Ntrk2*$^{lox/lox}$ (mutant) mice. n = 4 controls and 5 mutants. Two-tailed unpaired *t* test; n.s. = not significant, **P* = 0.0218, and ****P* = 0.0004. Error bars indicate SEM. Source data are provided as a Source Data file.

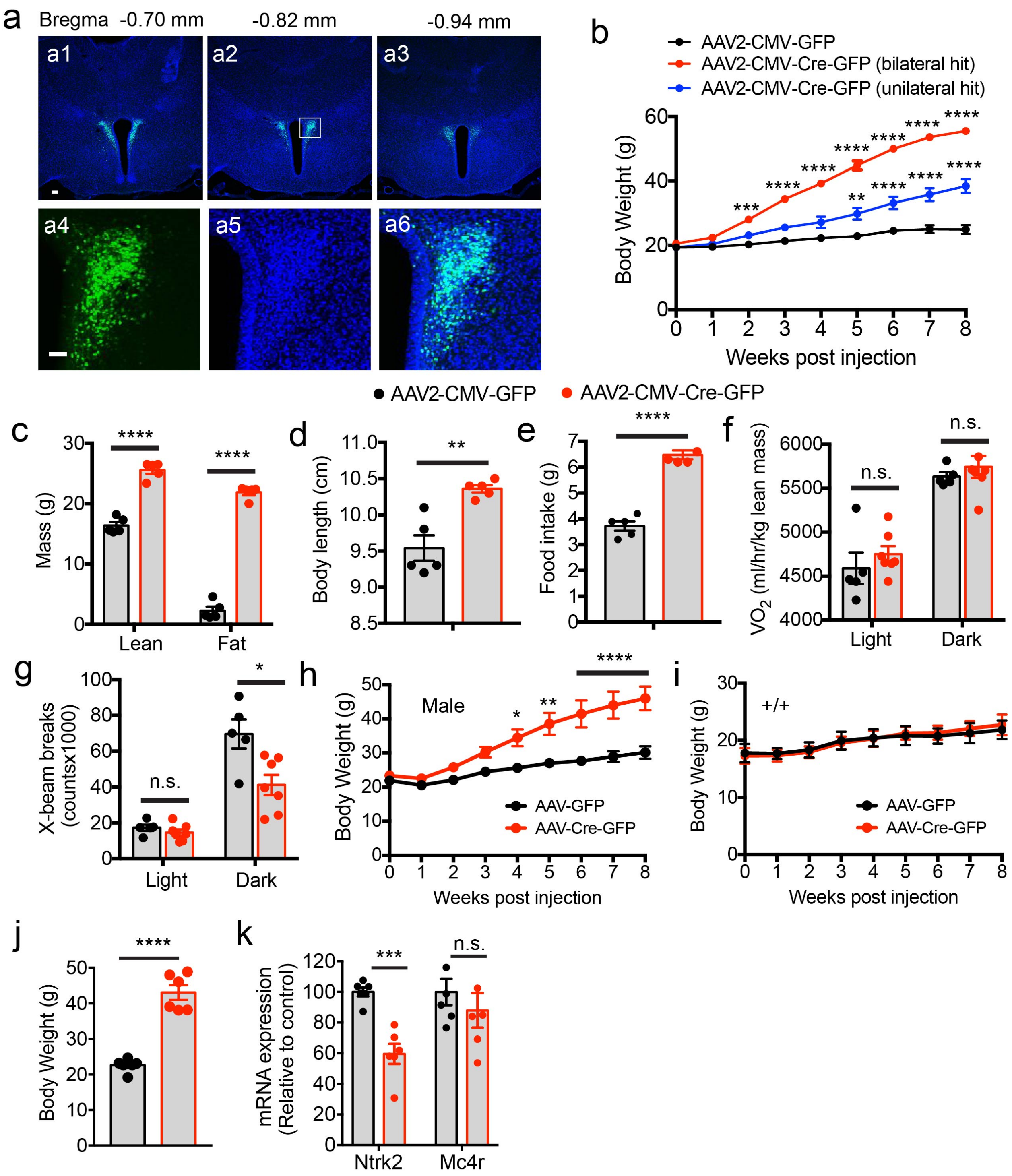

Supplementary Figure 4. Deletion of the *Ntrk2* gene in the PVH of adult mice leads to hyperphagic obesity. (**a**) Confocal images showing bilateral injection of AAV2-CMV-Cre-GFP into the PVH of female *Ntrk2*$^{lox/lox}$ mice (a1-a3) and intactness of the PVH after 9 weeks of AAV transduction (a4 – a6). Scale bar is 50 μm long. (**b**) Body weight of female *Ntrk2*$^{lox/lox}$ mice injected with either AAV2-CMV-GFP or AAV2-CMV-Cre-GFP (100 nl) into the PVH bilaterally.

Mice injected with AAV2-CMV-Cre-GFP are divided into two groups on the basis of either bilateral or unilateral hit. n = 5 mice for each group. Two-way ANOVA with post hoc Bonferroni multiple comparisons; $F_{(2, 108)}$ = 488.0, P < 0.0001 for viral injection; **P < 0.01, ***P < 0.001 and ****P < 0.0001 when compared with the AAV2-CMV-GFP group. (**c**) Body composition of female *Ntrk2*$^{lox/lox}$ mice 8 weeks after AAV injection. n = 5 mice for each group. Two-tailed unpaired *t* test; ****P < 0.0001. (**d**) Body length of female *Ntrk2*$^{lox/lox}$ mice 8 weeks after AAV injection. n = 5 mice for each group. Two-tailed unpaired *t* test; **P = 0.002. (**e**) Daily food intake of female *Ntrk2*$^{lox/lox}$ mice during post-injection week 4. n = 5 mice for each group. Two-tailed unpaired *t* test; ****P < 0.0001. (**f**) O_2 consumption of female *Ntrk2*$^{lox/lox}$ mice during post-injection week 4. n = 5 mice for AAV2-CMV-GFP and 7 mice for AAV2-CMV-Cre-GFP. Two-tailed unpaired *t* test; n.s., not significant. (**g**) Locomotor activity of female *Ntrk2*$^{lox/lox}$ mice during post-injection week 4. n = 5 mice for AAV2-CMV-GFP and 7 mice for AAV2-CMV-Cre-GFP. Two-tailed unpaired *t* test; n.s. = not significant and *P = 0.014. (**h**) Body weight of male *Ntrk2*$^{lox/lox}$ mice injected with either AAV2-CMV-GFP or AAV2-CMV-Cre-GFP into the PVH bilaterally. n = 6 mice for AAV2-CMV-GFP and 7 mice for AAV2-CMV-Cre-GFP. Two-way ANOVA with post hoc Bonferroni multiple comparisons; $F_{(1, 90)}$ = 80.53, P < 0.0001 for viral injection; *P = 0.0268, **P = 0.0013, and ***P < 0.0001. (**i**) Body weight of female WT mice injected with either AAV2-CMV-GFP or AAV2-CMV-Cre-GFP into the PVH bilaterally. n = 3 mice for AAV2-CMV-GFP and 7 mice for AAV2-CMV-Cre-GFP. Two-way ANOVA with post hoc Bonferroni multiple comparisons; $F_{(1, 64)}$ = 0.01326, P = 0.9112 for viral injection. (**j**) Body weight of female *Ntrk2*$^{lox/lox}$ mice injected with either AAV2-CMV-GFP or AAV2-CMV-Cre-GFP into the PVH bilaterally. Body weights were measured 8 weeks after AAV injection. These mice were used for measurement of *Mc4r* mRNA. n = 6 mice per group. Two-tailed unpaired *t* test; ****P < 0.0001. (**k**) Relative levels of *Ntrk2* mRNA and *Mc4r* mRNA in the PVH of the mice described in (j). n = 6 mice per group. Two-tailed unpaired *t* test; n.s. = not significant and ***P = 0.0002. Error bars indicate SEM. Source data are provided as a Source Data file.

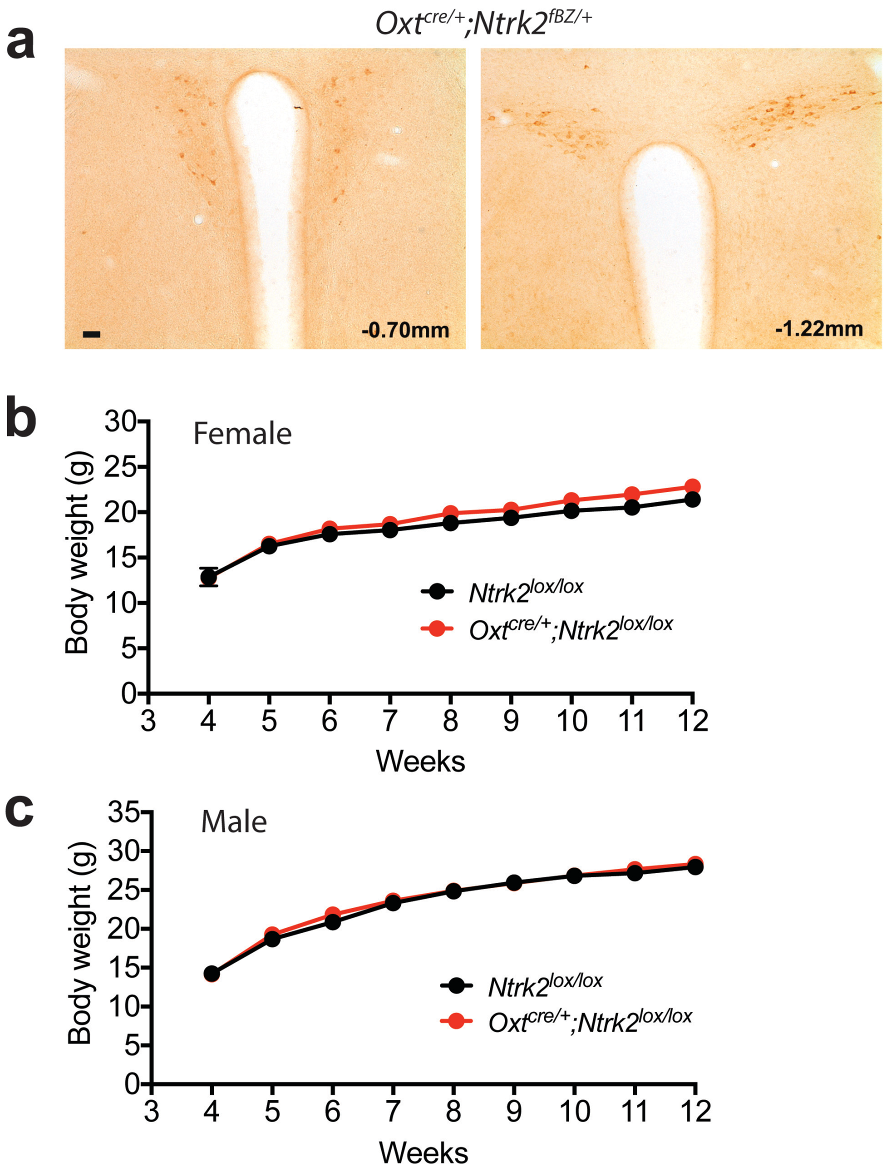

Supplementary Figure 5. Deletion of the *Ntrk2* gene in oxytocin-expressing cells does not alter body weight. (**a**) β-galactosidase immunohistochemistry on *Oxt*$^{Cre/+}$;*Ntrk2*$^{fBZ/+}$ brain sections. The approximate locations of the two brain sections relative to the bregma are indicated. Scale bar is 50 μm long. (**b**) Body weight of female *Ntrk2*$^{lox/lox}$ (control) and *Oxt*$^{Cre/+}$;*Ntrk2*$^{lox/lox}$ (mutant) mice. n = 6 controls and 8 mutants. Two-way ANOVA with post hoc Bonferroni multiple comparisons; $F_{(1,\ 96)}$ = 1.482, P = 0.2468 for genotype. (**c**) Body weight of male *Ntrk2*$^{lox/lox}$ and *Oxt*$^{Cre/+}$;*Ntrk2*$^{lox/lox}$ mice. n = 9 controls and 8 mutants.Two-way ANOVA with post hoc Bonferroni multiple comparisons; $F_{(1,\ 120)}$ = 3.256, P = 0.6477 for genotype. Error bars indicate SEM. Source data are provided as a Source Data file.

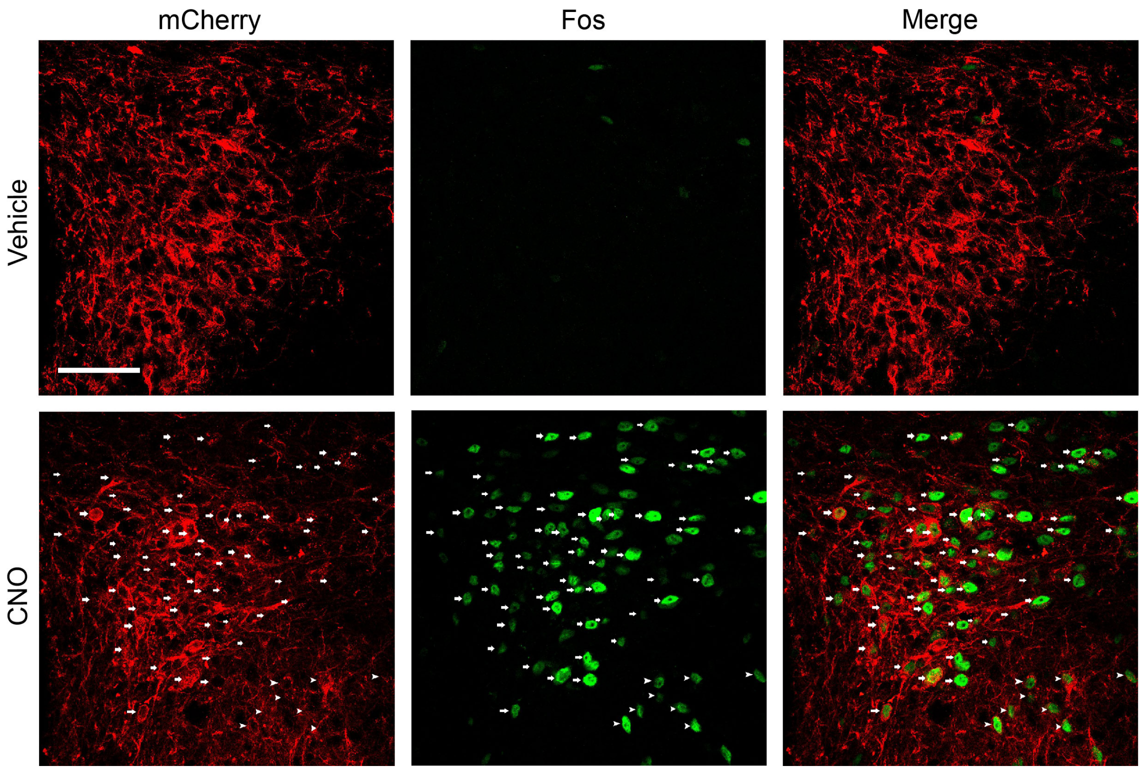

Supplementary Figure 6. CNO administration induces Fos expression in hM3D(Gq)-expressing PVH cells. *Ntrk2*$^{CreER/+}$ mice that express hM3D(Gq)-mCherry in PVH TrkB cells were treated with either vehicle or CNO and killed two hours after the treatment. Brain sections were used for examination of mCherry expression and Fos induction in the PVH. White arrows denote cells that express both mCherry and Fos. Scale bar, 50 μm.

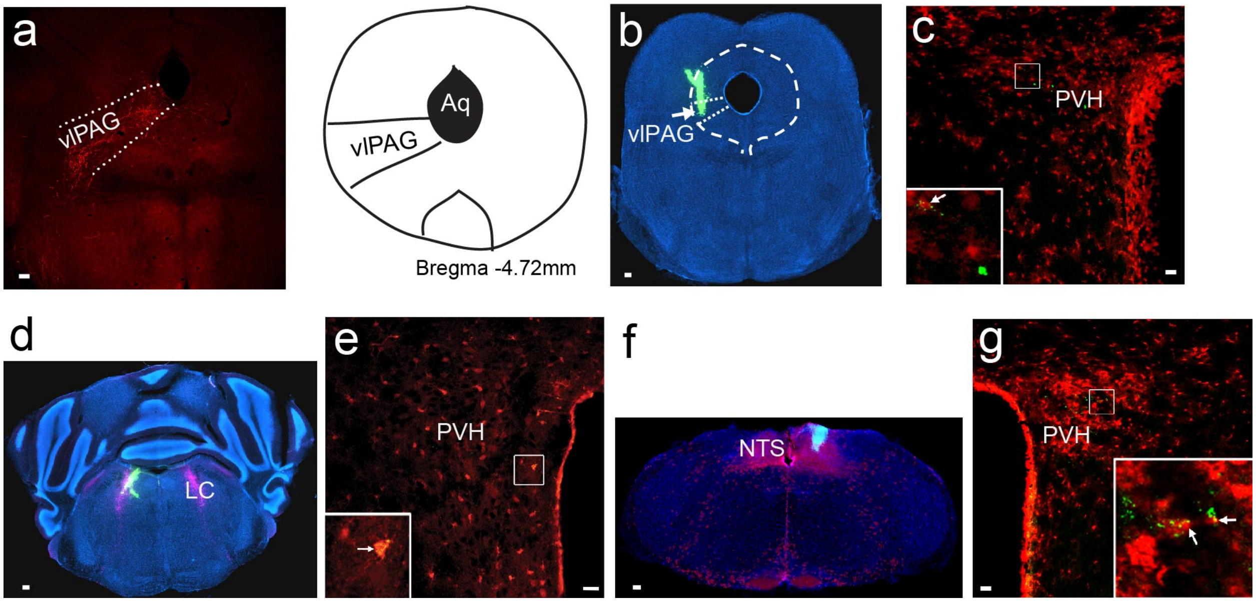

Supplementary Figure 7. Projections of PVH^{TrkB} neurons. (**a**) AAV2-CAG-FLEX-tdTomato was injected into the PVH of *Ntrk2*$^{CreER/+}$ mice. After the mice were treated with tamoxifen, tdTomato-labeled axonal terminals were detected in the ventrolateral periaqueductal gray (vlPAG). (**b, c**) Green retrobeads (GRBs) were unilaterally injected into the vlPAG of tamoxifen-treated *Ntrk2*$^{CreER/+}$*;Rosa26*$^{Ai9/+}$ mice (b), and GRBs were detected in some tdTomato-labeled PVH^{TrkB} neurons (c). (**d, e**) GRBs were unilaterally injected into the locus coeruleus (LC) of tamoxifen-treated *Ntrk2*$^{CreER/+}$*;Rosa26*$^{Ai9/+}$ mice (d), and GRBs were detected in some PVH^{TrkB} neurons (e). The LC is marked by tyrosine hydroxylase immunoreactivity (pink). (**f, g**) GRBs were unilaterally injected into the nucleus tractus solitaris (NTS) of tamoxifen-treated *Ntrk2*$^{CreER/+}$*;Rosa26*$^{Ai9/+}$ mice (f), and GRBs were detected in some PVH^{TrkB} neurons (g). The scale bars represent 100 μm in (a, b, d, f) and 50 μm in other panels.

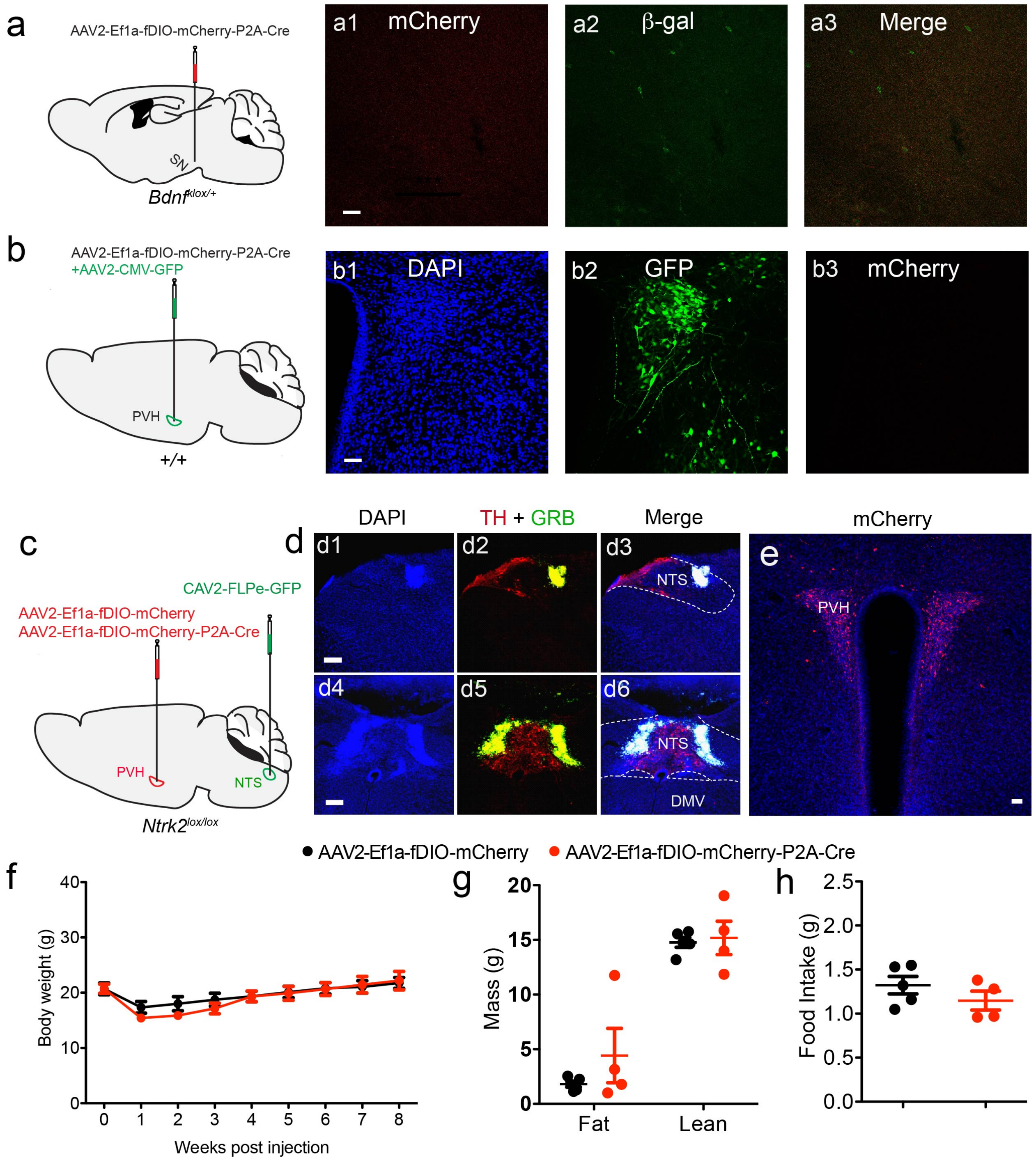

Figure 8. Deletion of the *Ntrk2* gene in PVH neurons projecting to the NTS. (**a**) Injection of AAV2-Ef1a-fDIO-mCherry-P2A-Cre (200 nl) alone into the substantia nigra did not lead to expression of mCherry and β-galactosidase (β-gal). (**b**) Injection of AAV2-Ef1a-fDIO-mCherry-P2A-Cre (150 nl) into the PVH did not lead to expression of mCherry-P2A-Cre. AAV-GFP (50 nl) was co-injected into the brain to mark the injection site. (**c**) Deletion of the *Ntrk2* gene in PVH neurons projecting to the NTS. A mixture of CAV2-FLPe + GRB (4:1 ratio) and either AAV2-Ef1a-fDIO-mCherry (control) or AAV2-Ef1a-fDIO-mCherry-P2A-Cre (150 nl per site) were

bilaterally injected into the NTS and PVH of *Ntrk2^lox/lox^* mice, respectively. Co-injected GRBs were used to mark CAV2 injection sites. The CAV2-FLPe + GRB mixture was injected into two sites for each NTS, a rostral site and a caudal site in the central NTS (100 nl per site). (**d**) CAV2 injection sites in the rostral part of central NTS (d1-3) and the caudal part of central NTS (d4-6). Tyrosine hydroxylase (TH) immunoreactivity (red) outlines the NTS, while GRBs (green) indicate CAV2 injection sites. (**e**) Neurons that were transduced by both CAV2-FLPe and AAV2-Ef1a-fDIO-mCherry-P2A-Cre expressed mCherry in the PVH. The image is a projection of several z optical sections. (**f-h**) Body weight (f), fat mass and lean mass (g), and 4-hour food intake (h) of female $Ntrk2^{lox/lox}$ mice injected with CAV2-FLPe into the NTS and either AAV2-Ef1a-fDIO-mCherry or AAV2-Ef1a-fDIO-mCherry-P2A-Cre into the PVH. Food intake was measured in the first 4 hours of the dark cycle. Body weight data were analyzed using two-way ANOVA with Bonferroni *post hoc* tests; $F_{(1, 96)} = 0.1720$, $P = 0.6856$ for virus (n=6 mice for control and 8 mice for Cre). Two-tailed unpaired *t* test reveals no significant difference in body composition and food intake between groups (n=5 mice for control and 4 mice for Cre). Scale bar in (d) represents 100 μm and in other panels 50 μm. Error bars indicate SEM. Source data are provided as a Source Data file.

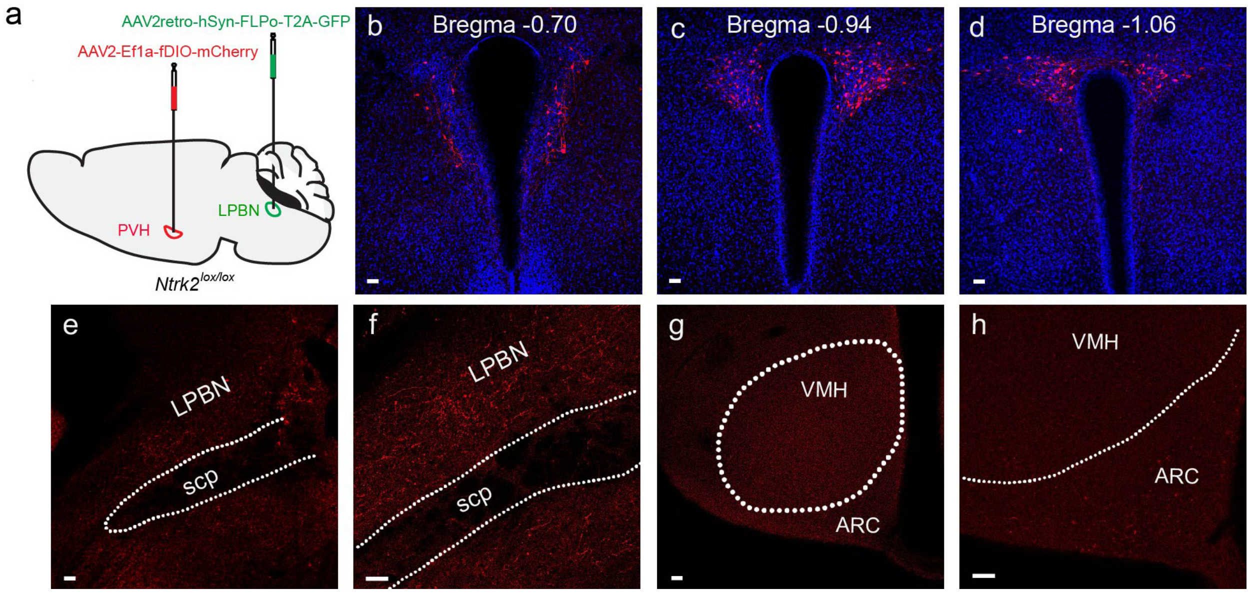

Supplementary Figure 9. Axonal terminals of PVH neurons projecting to the LPBN. (**a**) AAV2-Ef1a-fDIO-mCherry and AAV2retro-hSyn-FLPo-T2A-GFP were injected into the PVH and the LPBN of *Ntrk2*$^{lox/lox}$ mice, respectively. (**b**-**d**) Expression of mCherry in the PVH at various rostral-caudal positions. (**e**-**h**) mCherry-labeled axonal terminals in the LPBN and the VMH. ARC, arcuate nucleus; LPBN, lateral parabrachial nucleus; scp, superior cerebellar peduncle; VMH, ventromedial hypothalamus. Scale bars represent 50 μm.

Supplementary Table 1. Infection scores for AAV-Cre-GFP injected female *Ntrk2*$^{lox/lox}$ mice

			Female AAV-Cre Bilateral PVH Scores & Non-PVH Total Scores										
			Missed					Hit					
PVH	Anterior	**dorsal:**	+	-	+	-	+	-	+	-	++	++++	++++
		**ventral:**	+	-	-	-	++	-	-	-	-	+++	+++
	Central	**dorsomedial:**	-	-	-	+	-	+	-	++++	++++	++++	+++++
		**dorsolateral:**	-	-	+	+	-	+	+++	++++	+++++	++++	+++++
		**ventral:**	-	-	-	-	+	++	+	+++	++	+++	++++
	Posterior	**medial:**	-	+	-	+	-	+++	++	++++	++	+++	+++
		**lateral:**	-	+	-	-	-	+	+	+	+	+	+
		**Overall Bilateral PVH Score:**	**2**	**2**	**2**	**3**	**4**	**8**	**8**	**16**	**16**	**22**	**25**
Non-PVH Hypothalamus		Anterior (AH):	-	-	-	-	••	•	•	•	-	-	-
		Dorsmedial (DMH):	-	•	-	-	-	•	-	•	-	-	-
		Ventromedial (VMH):	-	•	-	-	-	-	•	•	-	-	-
		Lateral (LH):	-	-	-	-	-	-	-	-	-	-	-
		Arcuate (ARC):	-	-	-	-	-	-	-	-	-	-	-
		Posterior (PH):	-	••	-	••	-	-	-	-	-	-	•
		Suprachiasmatic (SCN):	-	-	-	-	-	-	-	-	-	-	-
		Medial Preoptic (MPO):	••	-	-	-	••	-	•	-	-	-	-
		Median Preoptic (MnPO):	•	-	-	-	-	-	-	-	-	-	-
		Zona Incerta:	-	•	•	-	•	•	•	•	•	•	•
		Subincertal Nucleus:	-	-	-	•	-	•	-	-	•	-	•
		Bed Nucleus of Stria Terminalis:	•	-	-	-	•	-	-	-	-	-	-
THALAMUS		Mediodorsal (MD):	•	•••	••	••••	-	•••	••	••	••	••	-
		Paratenial (PT):	••••	-	••	-	••	••	-	-	••	••	-
		Submedius (Sub):	-	•	-	••	-	•	-	••	•	••	-
		Reuniens (Re):	-	•	-	••	-	-	•	•	•	•	•
		Anteromedial (AM):	-	-	-	•••	-	••	••	-	-	-	-
		Paraventricular (PVT):	••	-	-	-	••	••	•	-	-	-	-
		Interanteromedial (IAM):	-	-	-	•	-	-	-	-	•	•	••
		Rhomboid (Rh):	-	-	-	•••	-	-	-	-	-	-	-
		Central Medial (CM):	-	-	-	••	-	-	-	-	-	-	-
		Interanterodorsal (IAD):	-	-	-	-	-	-	-	-	-	-	•
		Ventromedial (VM):	-	-	-	•	-	-	-	-	-	-	-
		Habenula:	-	•	-	•	-	-	-	-	-	-	-
		Hippocampus:	-	•	-	•	-	-	-	-	-	-	-
		Cingulate/Motor Cortex:	-	•	-	•	•	-	•	-	-	-	-
