# Supplementary material for: TrkB-expressing paraventricular hypothalamic neurons suppress appetite through multiple neurocircuits
Source: Nat Commun. 2020 Apr 7;11:1729. doi: 10.1038/s41467-020-15537-w (PMC7138837; doi:10.1038/s41467-020-15537-w)
Supplement: Supplementary file 3 — Reporting Summary [file 41467_2020_15537_MOESM3_ESM.pdf]

nature research

Corresponding author(s): Baoji Xu

Last updated by author(s): Feb 11, 2020

Reporting Summary

Nature Research wishes to improve the reproducibility of the work that we publish. This form provides structure for consistency and transparency in reporting. For further information on Nature Research policies, see Authors & Referees and the Editorial Policy Checklist .

Statistics

For all statistical analyses, confirm that the following items are present in the figure legend, table legend, main text, or Methods section.

n/a	Confirmed	
[ ]	[x]	The exact sample size (*n*) for each experimental group/condition, given as a discrete number and unit of measurement
[ ]	[x]	A statement on whether measurements were taken from distinct samples or whether the same sample was measured repeatedly
[ ]	[x]	The statistical test(s) used AND whether they are one- or two-sided<br>*Only common tests should be described solely by name; describe more complex techniques in the Methods section.*
[ ]	[x]	A description of all covariates tested
[x]	[ ]	A description of any assumptions or corrections, such as tests of normality and adjustment for multiple comparisons
[ ]	[x]	A full description of the statistical parameters including central tendency (e.g. means) or other basic estimates (e.g. regression coefficient) AND variation (e.g. standard deviation) or associated estimates of uncertainty (e.g. confidence intervals)
[ ]	[x]	For null hypothesis testing, the test statistic (e.g. *F*, *t*, *r*) with confidence intervals, effect sizes, degrees of freedom and *P* value noted<br>*Give P values as exact values whenever suitable.*
[x]	[ ]	For Bayesian analysis, information on the choice of priors and Markov chain Monte Carlo settings
[x]	[ ]	For hierarchical and complex designs, identification of the appropriate level for tests and full reporting of outcomes
[x]	[ ]	Estimates of effect sizes (e.g. Cohen's *d*, Pearson's *r*), indicating how they were calculated

Our web collection on statistics for biologists contains articles on many of the points above.

Software and code

Policy information about availability of computer code

Data collection	LAS AF (Immunoflourescence); NIS-Elements Confocal (Immunoflourescence); minispec mq (body composition); oxymax (oxygen consumption and locomotor activity), StepOne software (quantitative real time PCR)
Data analysis	ImageJ (v.1.50), GraphPad Prism 7

For manuscripts utilizing custom algorithms or software that are central to the research but not yet described in published literature, software must be made available to editors/reviewers. We strongly encourage code deposition in a community repository (e.g. GitHub). See the Nature Research guidelines for submitting code & software for further information.

Data

Policy information about availability of data

All manuscripts must include a data availability statement. This statement should provide the following information, where applicable:

- Accession codes, unique identifiers, or web links for publicly available datasets
- A list of figures that have associated raw data
- A description of any restrictions on data availability

The authors will make all data available to readers upon reasonable request.

Field-specific reporting

Please select the one below that is the best fit for your research. If you are not sure, read the appropriate sections before making your selection.

[x] Life sciences [ ] Behavioural & social sciences [ ] Ecological, evolutionary & environmental sciences

Life sciences study design

All studies must disclose on these points even when the disclosure is negative.

Sample size	No statistical methods were used to pre-determine sample sizes. We determined sample sizes on the basis of our previous studies and similar publications.
Data exclusions	In virus injection experiments, mice with missed injections (low or no viral expression at the target site were either assigned to a separate group or excluded in final data analyses.
Replication	In experiments with groups of mice, a sufficient number of mice in each group were used. Viral gene deletion experiments were repeated more than once using a separate batch of mice, and data were then combined for statistical analysis. Experiments examining co-localization of markers used one or two mice for each marker. Two to four mice were used to confirm Ntrk2 deletion in the PVH using in situ hybridization. Analysis of axonal projections was repeated in multiple mice.
Randomization	In virus-mediated gene deletion experiments and DREADDs experiments, mice with similar body weight were randomly assigned to different groups.
Blinding	Wherever possible (i.e. oxygen consumption, examination of viral injection site, food intake measurement in DREADDs-expressing mice, Fos induction in PVH-VMH and PVH-LPBN TrkB neurons after refeeding), analyses were performed by blinded observers and/or software-automated analyses.

Reporting for specific materials, systems and methods

We require information from authors about some types of materials, experimental systems and methods used in many studies. Here, indicate whether each material, system or method listed is relevant to your study. If you are not sure if a list item applies to your research, read the appropriate section before selecting a response.

Materials & experimental systems

n/a	Involved in the study	
☐	☒	Antibodies
☒	☐	Eukaryotic cell lines
☒	☐	Palaeontology
☐	☒	Animals and other organisms
☒	☐	Human research participants
☒	☐	Clinical data

Methods

n/a	Involved in the study	
☒	☐	ChIP-seq
☒	☐	Flow cytometry
☒	☐	MRI-based neuroimaging

Antibodies

Antibodies used	chicken anti-β-galactosidase (1:3,000; abcam #ab9361), rabbit anti-NeuN (1:1,000; Millipore #ABN78), rabbit anti-GFAP (1:400; Sigma #G9269), mouse anti-oxytocin (1:5,000; Millipore #MAB5296), rabbit anti-CRH (1:500; Millipore #AB1760), mouse anti-GFP (1:1,000; Clontech #632460), rabbit anti-somatostatin (1:500; immunostar #20067), rabbit anti-GHRH (1:500; Immunostar #22938), rabbit anti-vasopressin (1:5,000; Millipore #AB1565), mouse anti-tyrosine hydroxylase (1:10,000; Sigma #T1299), rabbit anti-c-Fos antibody (1:5,000; abcam #ab208942), rabbit anti-prodynorphin (1:200; abcam#ab11137), rabbit anti-DsRed (1:1000; Takara #632496), and rabbit anti-TRH (1:10,000; generous gift from Dr. M. Wessendorf , University of Minnesota).
Validation	All antibodies except anti-TRH are commercially available. We validated the antibodies using negative tissues and/or expected expression patterns.

Animals and other organisms

Policy information about studies involving animals; ARRIVE guidelines recommended for reporting animal research

Laboratory animals	Floxed Ntrk2 (Ntrk2lox; also known as TrkBlox, floxed Ntrk2-LacZ (Ntrk2fBZ; also known as fBZ) were generated in our lab as previously described. Floxed Bdnf (Bdnf klox) mouse strains was provided by Dr. Kevin R Jones at University of Colorado Boulder. Sim1-Cre (stock No: 006395), MC4R-tau-GFP (stock No: 008323), Rosa26Ai9 (Gt9(ROSA)26Sor tm99CAG-tdTomato)Hze/J; stock No: 007909) and C57BL6/J (stock No: 000664) mouse strains were obtained from the Jackson Laboratory. The Ntrk2CreER/+ (also known as TrkBCreER) mouse strain33 was kindly provided by Dr. David Ginty at Harvard Medical School.
Wild animals	C57BL/6J mice were obtained from Jackson Laboratory
Field-collected samples	This study did not involve samples collected from the field.

Ethics oversight	Animal care and experimental procedures were approved by Scripps Florida Institutional Animal Care and Use Committee (protocol # 16-003), according to US National Institutes of Health Guidelines (Online Methods/mouse husbandry).

Note that full information on the approval of the study protocol must also be provided in the manuscript.
